# Is social connection the key to prevention? Educational attainment, incident dementia, and the role of later-life social engagement

**DOI:** 10.1093/geroni/igag051

**Published:** 2026-05-04

**Authors:** Alyssa W Goldman, Hyungmin Cha, Reza Tayari Ashtiani

**Affiliations:** Department of Sociology, Boston College, Chestnut Hill, Massachusetts, United States; Department of Sociology, Center for Studies in Demography and Ecology, University of Washington, Seattle, Washington, United States; Department of Sociology, Boston College, Chestnut Hill, Massachusetts, United States

**Keywords:** Cognitive function, Volunteering, Social activities, Social network, Socioeconomic status

## Abstract

**Background and Objectives:**

Educational attainment and mid-to-later-life social engagement are modifiable risk factors for dementia, yet limited research examines how these factors intersect to shape dementia risk. This study estimates the extent to which social engagement may act as a pathway through which education influences incident dementia, and which forms of social engagement may most reduce educational differences in incident dementia among older adults in the United States.

**Research Design and Methods:**

We used survival and counterfactual decomposition analyses of the 2011–2018 National Health and Aging Trends Study to assess whether, and to what extent, different forms of social engagement mediate and interact with the effect of educational attainment on incident dementia. We compared total and controlled direct effects to assess how educational differences in incident dementia may shift under hypothetical scenarios of having large discussion networks and prior-month engagement in social activities among older adults with higher and lower levels of education.

**Results:**

Going out for enjoyment, visiting with friends/family, volunteering, and organized group activities were significant partial mediating pathways in the association between education and incident dementia. When prior-month volunteering, organized group activity attendance, and a large network size were modeled as mediators, educational differences in incident dementia were attenuated and became statistically nonsignificant.

**Discussion and Implications:**

Findings suggest increasing older adults’ participation in volunteering and group activities, as well as larger discussion networks, may reduce educational disparities in dementia risk. Future research should investigate the mechanisms driving these results and whether they extend to other social activities not examined in this study.

Innovation and Translation Statement: Social connectedness is an underexplored pathway through which education influences dementia. We used longitudinal data from the 2011–2018 National Health and Aging Trends Study to examine how six different forms of social engagement mediate the relationship between educational attainment and incident dementia. Using survival and decomposition analyses in a counterfactual framework, we found that volunteering, organized group activity engagement, and large discussion networks were especially likely to reduce the magnitude and statistical significance of the effect of education on incident dementia among older adults. Community interventions that increase older adults’ access to these social activities may be especially important in reducing educational disparities in later-life dementia risk.

Educational attainment is increasingly recognized as a key dimension of dementia disparities, as well as a significant modifiable risk factor for reducing dementia. In U.S. samples, lower educational attainment during adolescence and young adulthood is linked to poorer cognitive abilities and higher dementia rates in older adulthood, as well as fewer cognitively healthy years and a younger modal age of dementia onset (Cha et al., 2024; Crimmins et al., 2018; Nguyen et al., 2016). Improvements in educational attainment in the United States are associated with downward trends in the prevalence and incidence of dementia since 2000 (Farina et al., 2022), including reductions in racial/ethnic disparities (Garcia et al., 2021; Hayward et al., 2021), and declines in age-specific dementia risks among later-born cohorts (Langa et al., 2017).

A prevailing view is that the association between education and dementia is partly attributed to the cognitively stimulating environment of education, which is believed to exert an enduring protective influence on cognitive abilities and dementia risk throughout the life span (Cha et al., 2025; Hayward et al., 2021; Tucker-Drob, 2019). Education functions as a foundational exposure that equips individuals with a stock of cognitive skills, strategies, and problem-solving abilities that can be drawn upon long after formal schooling ends. These accumulated resources shape how people process information, adapt to challenges, and navigate complex environments across the life course (Stern, 2009). Because dementia reflects a threshold—when cognitive abilities decline to levels that interfere with everyday functioning—education is consequential not by preventing decline altogether but by sustaining higher overall levels of ability (Lövdén et al., 2020; Tucker-Drob, 2019). As a result, individuals with more education are more likely to remain above the threshold for a longer period, thereby reducing their risk of being classified as having dementia at a given age.

Parallel research highlights the largely protective effects of social engagement in mid and later life. Larger, more diverse social networks and frequent engagement in social activities are consistently linked with better cognitive function and lower dementia risk (Ellwardt et al., 2015; Peng et al., 2022; Peng & Perry, 2025; Perry, Roth, et al., 2022). Networks that include connections with weakly connected individuals (i.e., bridging positions) are sources of cognitive stimulation, offering more social enrichment and increases to cognitive reserve than smaller, more homogenous social networks (Peng et al., 2022; Perry, Roth, et al., 2022). Social network connections and engagement in social activities can provide exposure to novel information through social interactions and draw individuals into diverse environmental contexts, which are also sources of enrichment, stimulation, and social support (Drabo et al., 2025; Ellwardt et al., 2015; Peng & Perry, 2025; Perry, McConnell, Coleman, et al., 2022). Notably, even among older adults with Alzheimer’s pathology, those with larger social networks demonstrated higher cognitive function (Bennett et al., 2006). In another study, personal network characteristics moderated the relationship between structural brain changes (e.g., atrophy) and cognitive function (Perry, McConnell, Coleman, et al., 2022).

Social engagement is also patterned by education, in ways that prompt deeper consideration of how these domains jointly shape dementia risk. Indeed, regional and national surveys indicate that higher educational attainment is associated with larger social networks (Ajrouch et al., 2005) and greater involvement in community organizations (Andersson, 2018), while lower education levels are associated with smaller networks, fewer non-kin ties, and less support from non-kin social network ties in later life (e.g., Andersson, 2018; Van Groenou & Van Tilburg, 2003). Educational experiences, especially college attendance, are important avenues for building social networks and social capital (e.g., Chetty et al., 2022). The interrelationships between education, social engagement, and dementia risk are less widely considered (c.f., Du et al., 2023), and may intersect through multiple conceptual pathways.

## Additive effects of education and social engagement

Education and social engagement in mid-to-later life may independently shape dementia risk. Indeed, studies identifying significant links between social engagement and cognitive function and dementia also observe associations between education and cognitive outcomes when included as predictors in the same models (e.g., Chen et al., 2025; Ellwardt et al., 2015; James et al., 2011). Life-long protective effects of education may be robust to midlife experiences, such as occupation, social engagement, and health risks (Cha et al., 2025). Likewise, cognitive benefits of social engagement, including cognitive stimulation and enrichment, as well as health correlates such as physical activity and health-promoting behaviors, may exist regardless of educational background.

## Mediating role of social engagement

Social engagement may be a mediating pathway through which education shapes dementia risk (Du et al., 2023). Cognitive benefits associated with education may be derived in part through greater access to social resources and higher levels of social engagement that are associated with higher socioeconomic status, including larger personal networks and greater involvement in organized social activities (e.g., Ajrouch et al., 2005; Young et al., 2025). Some hypotheses point to social engagement as a source of cognitive stimulation and enrichment that is associated with cognitive reserve (e.g., Ellwardt et al., 2015; Peng et al., 2022; Peng & Perry, 2025). Evidence indicates that socially stimulating network characteristics are associated with episodic memory and executive function (e.g., Perry, McConnell, Peng, et al., 2022), while social activity engagement, such as volunteering, is related to episodic memory, working memory, and verbal fluency (Sharifi et al., 2024). Other theories emphasize the role of social engagement in stress reduction and vascular processes that are associated with cognitive function and dementia (e.g., Fratiglioni et al., 2004). Collectively, these pathways suggest that social engagement may help to translate educational advantages into better cognitive outcomes. Indeed, one recent study estimates that social isolation may explain nearly 4% of the association between educational attainment and Montreal Cognitive Assessment scores among older adults (Goldman, Wroblewski, et al., 2025), although the mediating roles of specific forms of social engagement (e.g., specific social activities) have yet to be explored.

## Interactive effects of social engagement and education

The influence of educational attainment on dementia risk may be conditional, in part, on social engagement in older adulthood. More frequent patterns of social engagement may amplify the cognitive benefits of higher levels of educational attainment, providing additionally stimulating social environments for cognitive abilities to be in demand or “exercised” (Marioni et al., 2015; Staff et al., 2018). Other research finds a compensatory effect, whereby exposure to socially stimulating environments in later life reduces differences in cognitive function between older adults with low and high levels of education, exerting stronger protective effects among those with less than a high school degree (Peng & Perry, 2025). Indeed, social engagement may be especially beneficial for older adults with lower levels of education who may have gained fewer cognitive benefits from foundational education experiences compared to those with higher educational attainment earlier in the life course.

In this study, we begin to integrate the largely separate literatures linking education and social engagement with cognitive function by estimating both the mediating and interactive effects of different forms of social engagement on educational differences in incident dementia over 8 years. At present, this literature remains fragmented. Public health concerns about the burdens of dementia have increased alongside recent concerns about social isolation in the U.S., prompting efforts to increase social engagement, especially among older adults (e.g., Gryp et al., 2025; Office of the Surgeon General (OSG), 2023). Using a counterfactual framework with nationally representative data, our analyses shed light on a broader question: would later-life interventions designed to increase social engagement influence educational disparities in dementia risk?

## Data and methods

We use eight rounds of data from the National Health and Aging Trends Study (NHATS). The NHATS is a nationally representative longitudinal study of United States Medicare beneficiaries aged 65 and older and follows respondents as they transition to different residential contexts (community-dwelling, residential care places, nursing homes, and other assisted living facilities). Data collection is conducted through in-home interviews. Proxy interviews were administered in cases where the respondent was ill, impaired, or otherwise unable to complete the interview themselves. The NHATS began in 2011 (*N *= 8,245; 71% response rate) and has since administered annual re-interviews, with sample replenishments in 2015 and in 2022/2023. Our analysis uses data from respondents who began the study in 2011 (Round 1) and follows these respondents through 2018 (Round 8).

### Cognition and dementia

At each survey round, the NHATS administered a cognitive screening to all self-reporting respondents, assessing memory, executive function, and orientation. Proxy interviews were also asked to report on dementia and cognitive impairment of the sample person using the AD8 Screening Interview (Galvin et al., 2005). Based on the NHATS classification procedures (Kasper et al., 2013), respondents were categorized as having “probable dementia” if they scored 1.5 SDs or more below the sample mean on at least two cognitive test domains, reported a dementia diagnosis, or scored 2 or higher on the AD8. Our analyses use a more conservative definition of dementia (Kasper et al., 2013), treating those who screen positive for “possible dementia” in the NHATS as having “no dementia.”

### Educational attainment

Respondents reported their educational attainment as the highest degree completed as part of the Round 1 interview. We coded responses using four categories: less than a high school degree, high school degree or equivalent, some college or Associate’s degree, or Bachelor’s degree or higher.

### Social engagement

At each interview, NHATS respondents were asked if *in the last month* they had ever visited in person with friends or family not living with them, volunteered, participated in organized group activities, gone out for enjoyment, or attended religious services. We coded affirmative responses (“yes”) as 1 and negative responses (“no”) as 0. Self-reporting respondents were also asked: “Looking back over the last year, who are the people you talked with most often about important things?” Respondents could name up to 5 individuals. The total number of named individuals represents the core discussion *network size* (range: 0–5). We use social engagement measures taken from the baseline survey in our main analyses (Round 1, 2011). As education was completed earlier in the life course before respondents participated in the NHATS, the use of Round 1 social engagement measures to predict future incident dementia preserves the temporal ordering for mediation (Cole & Maxwell, 2003).

### Covariates

We adjusted all models for respondent age (categorical), gender, whether the respondent is Black, and whether the respondent is Hispanic, all measured at the Round 1 baseline survey. We also controlled for whether the respondent was married to or living with a partner and whether the respondent had worked for pay recently. Self-rated health was measured using a Likert scale asking respondents to rate their overall health, where 1 = “Excellent” and 5 = “Poor.” We reverse-coded responses so that higher scores indicated better overall health. Depression was assessed using the Patient Health Questionnaire-2, asking respondents to rate how often they “had little interest or pleasure in doing things” and “felt down, depressed, or hopeless” over the past month, where 0 = “not at all” and 3 = “nearly every day.” We summed responses to both questions and coded total scores of 3 or higher as screening positive for depression (Xiang, 2020). We also adjusted for difficulty with basic activities of daily living, coded as the sum of activities that respondents reported needing help with in the prior month (eating, dressing, bathing, toileting; range: 0 to 4). Current smoking status was measured dichotomously (1 = yes, currently smokes).

### Analysis

We used survival analysis within a counterfactual mediation framework to examine how higher levels of social engagement influence educational differences in incident dementia. Time-to-event is measured in years since 2011. Incident dementia is defined as the first wave from 2012 to 2018 in which a respondent screened as “probable dementia.” We use Cox regression models for consistency across the additive and counterfactual analyses, as the med4way package used for the decomposition analyses does not have built-in commands for discrete-time survival models. We acknowledge that the NHATS administers the dementia screening at the annual survey intervals, and we do not have more precise data on the timing of dementia incidence. (In sensitivity analyses, we conducted discrete-time survival analyses for the additive models, which yielded results consistent with those presented here). Censoring occurred if respondents never screened positive for dementia across the observation period, if they died prior to dementia onset, or due to non-response. Results were generally consistent when excluding people who died during the observation period.

The counterfactual framework allowed us to estimate how the effect of education on incident dementia might change under conditions in which a social engagement mediator is set to a specific value *m*. Widely used mediation approaches decompose the total effect (TE) of a predictor on the outcome into its direct and indirect components, with the indirect effect representing the portion of the relationship that is explained by the mediator (VanderWeele, 2014, 2016). The four-way decomposition approach allowed us to decompose the TE of education on incident dementia into the controlled direct effect (CDE), pure indirect effect, reference interaction (INT_ref_), and mediated interaction (INT_med_) (see Table 1 for notation and component interpretations). These estimates accounted for mediation, interaction, and mediated interaction. While we report all four components, we focus on the CDE, which captures the portion of the total effect of education that would remain under a hypothetical scenario in which all older adults had engaged in each social activity in the prior month (e.g., volunteering, organized group activities) and had large social networks (i.e., the maximum size of five network members) (VanderWeele, 2014, 2016).

**Table 1:** Summary of the components of the four-way decomposition of the total effect of education on incident dementia.

4-way decomposition	Counterfactual	Interpretation
**Total Effect (TE)**	Y_1_ –Y_0_	The overall effect of education on the relative risk of incident dementia, comparing those with a Bachelor’s degree or higher to those with less than a high school degree.
**Controlled Direct Effect (CDE)**	Y_11_ –Y_01_	The effect of education on incident dementia that would remain if the social engagement mediator were fixed at 1 (present) for all individuals, regardless of education.
**Reference Interaction (INT_ref_)**	(Y_11_ –Y_10_ –Y_01_ + Y_00_)(M_0_)	The portion of the total effect due to the interaction between education and social engagement when the social engagement mediator is set to the reference level (i.e., effect due to interaction only).
**Mediated Interaction (INT_med_)**	(Y_11_ –Y_10_ –Y_01_ + Y_00_)(M_1_ – M_0_)	The portion of the total effect due to both mediation and interaction, i.e., the effect of education operating through social engagement, combined with the interaction between education and social engagement (i.e., mediation and interaction).
**Pure Indirect Effect (PIE)**	(Y_01_ –Y_00_) (M_1_ –M_0_)	The effect of education on incident dementia that operates through the mediator (social engagement), and in the absence of exposure-mediator interaction (i.e., mediation only).

Note. Counterfactual notation follows VanderWeele (2014). Y_*a*_ refers to the potential outcome when education is set to *a*, where 1 = “Bachelor’s degree or more” and 0 = “less than high school.” Y_*am*_ refers to the potential outcome when education is *a* and the social engagement mediator is *m. M*_a_ refers to the potential value of the social engagement mediator if individual education is set to *a.* For network size, the mediator was fixed at 5 rather than 1. In our analysis, the outcome is modeled using Cox proportional hazards models, and the decomposition components are expressed as excess relative risks (ERRs).

In all decomposition analyses, educational attainment was the exposure variable, with estimated TEs and CDEs comparing a Bachelor’s degree or higher with less than a high school degree. Incident dementia was the outcome, defined as the first survey round in which the respondent’s cognitive assessment met the criteria for “probable dementia.” We conducted six sets of analyses to model each of the six social engagement mediators separately. First, a linear regression model was fit to estimate each social engagement mediator as a function of educational attainment and covariates. Second, a Cox proportional hazards model was fit to estimate the hazard of incident dementia as a function of the social engagement mediator, educational attainment, and the covariates, including an interaction term between education and social engagement. The TE of education on dementia was then decomposed into excess relative risks estimating the controlled direct effect, reference interaction, mediated interaction, and pure indirect effect, using the parameters from both models, with standard errors calculated using the delta method. All decomposition analyses were conducted using the *med4way* command in Stata 18 (Discacciati et al., 2019) and used respondent-level baseline sampling weights.

### Analytic sample and approach

We first examined descriptive statistics and bivariate patterns in dementia by levels of education and social engagement. Next, we used Cox proportional hazards models to examine the associations of education, each form of social engagement, and covariates with the hazard of incident dementia. We then used decomposition analysis to estimate the mediating and interactive effects of each social engagement mediator, as well as the controlled direct effects, that is, the effect of education on incident dementia if all respondents had high levels of social engagement.

In 2011 (Round 1), 7,609 NHATS respondents had non-missing values on dementia. Of these, we limited our analytic sample to the 6,461 NHATS respondents who did *not* screen positive for “probable dementia” at baseline (2011) and who had non-missing values on covariates. Less than 2% of cases are excluded due to missing data on covariates. Final sample sizes for each of the six sets of analyses vary slightly based on different missing data patterns across the social engagement mediators. These variables have missing values for less than 1% of cases, except for social network size, which is missing for approximately 1.9% of respondents, as this question module was not included in proxy interviews.

## Results

### Descriptive statistics

Table 2 includes descriptive statistics of the key variables in the analytic sample. Approximately 15% of NHATS respondents who were dementia-free at Round 1 screened positive for “probable dementia” at some point in the observation period (2012–2018). Educational attainment is relatively evenly distributed within the sample, with 24% having less than a high school education, 28% having a high school degree or equivalent, 26% having some college education, and 23% having a Bachelor’s degree or higher. On average, NHATS respondents reported having approximately two people in their core discussion networks at Round 1. Most NHATS respondents reported having visited with friends or family and gone out for enjoyment in the prior month (87% and 77%, respectively). Just 25% volunteered in the prior month, with 38% and 60% engaged in organized group activities and having attended religious services in the prior month, respectively.

**Table 2:** Descriptive statistics of key variables used in the analyses from the NHATS (*N *= 6,461).

Variable	Proportion	Mean (*SD*)
**Probable dementia** ^a^	.15	
**Education**		
Less than high school	.24	
High school or equivalent	.28	
Some college	.26	
Bachelor’s degree or higher	.23	
**Social engagement**		
Network size		1.99 (1.31)
“In the past month did you…:”		
Visit friends/family not living with them (1 = yes)	.87	
Go out for enjoyment (1 = yes)	.77	
Volunteer (1 = yes)	.25	
Organized group activities (1 = yes)	.38	
Religious service attendance (1 = yes)	.60	
**Covariates**		
Age (years)	**—**	
65–69	.21	
70–74	.23	
75–79	.20	
80–84	.19	
85–89	.11	
90+	.06	
Black	.21	
Hispanic or Latino/a	.05	
Female	.57	
Married or living with a partner	.52	
Worked for pay recently	.12	
Self-rated physical health		3.35 (1.08)
Currently smokes	.08	
Depressive symptoms		.12 (.33)
Basic activities of daily living		.14 (.51)

Note. NHATS = National Health and Aging Trends Survey. Means are weighted using NHATS person-level weights and adjusted for the NHATS survey design. Proportions are unweighted. Means and proportions are calculated from respondents who did not screen positive for probable dementia at Round 1 (2011), and who had non-missing data on Round 1 covariates.

a Calculated as proportion of respondents who ever screened positive for probable dementia from 2012-2018 (Kasper et al., 2013).

Survey-weighted descriptive comparisons indicated that social engagement is patterned by education. Indeed, 11% of older adults with less than a high school degree reported volunteering in the prior month, compared to 41% of those with a Bachelor’s degree (post-estimation Wald test: *t *= 17.40, *p* < .001). Approximately 20% of those with less than a high school degree participated in organized group activities in the prior month, compared to 58% of those with a Bachelor’s degree (*t *= 17.49, *p* < .001). Whereas the vast majority of those with a Bachelor's degree went out for enjoyment (91%), a smaller majority (63%) of those with less than a high school degree participated in this form of social engagement in the prior month (*t *= 13.95, *p* < .001). Most respondents across all levels of education reported visiting in person with friends and family, though differences in the proportions between those with a Bachelor’s and those with less than a high school degree were still significant (.93 vs. .81; *t *= 6.93, *p* < .001). We also observed larger average network sizes as educational attainment increases, with 2.24 network members among those with a Bachelor’s degree and 1.65 network members, on average, among those with less than a high school degree (*t *= 9.31, *p* < .001). Those with less than a high school degree included a lower proportion of respondents who attended religious services in the prior month (51%) compared to those with a Bachelor’s degree or higher (61%; *t *= 4.29, *p* < .001). Supplementary Figure 1 illustrates these differences by education.

In general, we find that respondents who reported social engagement in the prior month were less likely to screen positive for dementia. For example, 13% of those who reported that they did not volunteer in the prior month at Round 1 screened positive for probable dementia at some point during the observation period compared to 8% of those who did report volunteering in the prior month (*t *= 5.61, *p* < .001). Similar patterns and significant differences by incident dementia are observed across all forms of social engagement examined in this study, including network size. Consistent with prior studies, dementia is also patterned by education. Among NHATS respondents who were dementia-free at Round 1, 8.5% of those with a Bachelor’s degree screened positive for dementia by 2018, compared to 9.3% of those with “some college,” 11.7% of those with a high school degree or equivalent, and 21.2% of those with less than a high school degree. Supplementary Figure 2 illustrates the proportion of respondents who ever screened positive for probable dementia during the observation period by levels of social engagement at Round 1 (2011).

### Cox proportional hazards models

Table 3 presents hazard ratios from a series of Cox proportional hazards models that examined how each of the six social engagement mediators predicted incident dementia (Models 1–6), as well as collectively (Model 7). Each model adjusted for educational attainment and all covariates, estimating the additive effects of social engagement and education. Across all models, higher levels of education were associated with significantly lower hazards of dementia compared to older adults with less than a high school degree. Discussion network sizes of 3 and 4 were each associated with significantly lower hazards compared to those with 0 network members (Model 1), as was visiting friends/family (hazard ratio [HR] = .68, *p <* .001), going out for enjoyment (HR = .73, *p* < .001), volunteering (HR = .72, *p* < .001), and participating in organized group activities (HR = .83, *p* < .05) in the prior month. Model 7 indicates that when all social engagement measures are included, visiting family/friends (HR = .76, *p* < .01), going out for enjoyment (HR = .82, *p* < .05), and volunteering (HR = .78, *p* < .05), as well as a network size of 4 (HR = .66, *p* < .05), remain associated with significantly lower hazards of dementia.

**Table 3:** Hazard ratios from Cox proportional hazards models predicting incident dementia, examining additive effects of education and social engagement measures.

Variable	Model 1	Model 2	Model 3	Model 4	Model 5	Model 6	Model 7
**Education (*reference* = Less than high school)**							
High school or equivalent	0.72***	0.70***	0.71***	0.70***	0.70***	0.69***	0.75**
	(0.07)	(0.07)	(0.07)	(0.07)	(0.07)	(0.07)	(0.07)
Some college	0.62***	0.61***	0.62***	0.62***	0.62***	0.60***	0.66***
	(0.06)	(0.06)	(0.06)	(0.06)	(0.06)	(0.06)	(0.07)
Bachelor’s or more	0.62***	0.59***	0.61***	0.62***	0.61***	0.58***	0.69**
	(0.07)	(0.07)	(0.07)	(0.07)	(0.07)	(0.07)	(0.08)
**Network size (*reference *= 0)**							
1	0.88						0.93
	(0.13)						(0.14)
2	0.81						0.88
	(0.13)						(0.14)
3	0.67*						0.74
	(0.12)						(0.13)
4	0.59*						0.66*
	(0.13)						(0.14)
5	0.66						0.75
	(0.15)						(0.17)
**“In the past month did you…:”**							
Visit friends/family		0.68***					0.76**
		(0.06)					(0.07)
Go out for enjoyment			0.73***				0.82*
			(0.06)				(0.07)
Volunteer				0.72***			0.78*
				(0.07)			(0.08)
Organized group activities					0.83*		0.95
					(0.07)		(0.08)
Religious service attendance						0.87	0.97
						(0.07)	(0.08)
**Wald χ^2^ (df)**	908.72*** (25)	912.01*** (21)	906.71*** (21)	900.12*** (21)	902.43*** (21)	901.58*** (21)	913.56*** (30)
***N***	6,339	6,459	6,458	6,461	6,461	6,461	6,334

Note. Data are from the National Health and Aging Trends Survey (2012–2018). All models adjust for the full set of control variables shown in Table 2. Robust standard errors appear in parentheses.

*** *p* < .001; **p < .01; *p < .05 (two-sided tests).

### Total effect decomposition

Table 4 presents the four-way decomposition of the TE of education on incident dementia, examining the mediating and interactive effects of each of the six social engagement mediators. TEs represent the overall effect of education on incident dementia, expressed as excess relative risks, while CDEs represent the direct effect of education on incident dementia that would remain if all respondents had a network size of 5 and, separately, if participation in each social activity occurred in the prior month. Full results from the regression models underlying this decomposition are included in Supplementary Table 1.

**Table 4:** Results from six separate four-way decomposition analyses of the association between education and incident dementia, each examining a different social engagement mediator.

Mediator	Total effect (TE)	Controlled direct effect (CDE)	Reference interaction (INT_ref_)	Mediated interaction (INT_med_)	Pure indirect effect (PIE)	*N*
**Network size**	−.428*** (.067)	−.312 (.160)	−.087 (.169)	.013 (.030)	−.042 (.022)	6,339
**Visit friends/family**	−.437*** (.069)	−.405*** (.069)	−.022 (.034)	.006 (.008)	−.016* (.007)	6,459
**Go out for enjoyment**	−.442*** (.062)	−.404*** (.074)	.004 (.058)	−.001 (.028)	−.040* (.018)	6,458
**Volunteer**	−.444*** (.062)	−.144 (.141)	−.281 (.145)	.074 (.041)	−.093** (.032)	6,641
**Organized group activities**	−.440*** (.062)	−.221 (.126)	−.213 (.126)	.086 (.052)	−.092* (.038)	6,461
**Religious service attendance**	−.439*** (.062)	−.401*** (.088)	−.033 (.067)	.006 (.011)	−.011 (.009)	6,461

Note. Data are from the National Health and Aging Trends Survey (2012–2018). Estimates represent excess relative risks. Standard errors appear in parentheses.

*** *p* < .001; **p < .01; *p < .05 (two-sided tests).

The first column of Table 4 includes the TEs of higher education on incident dementia. Across all models, having a Bachelor’s degree or higher was associated with a significantly lower hazard of incident dementia compared to less than a high school degree (TE *p* < .001 in all models), regardless of which social engagement mediator is considered. The second column shows the CDEs. When social engagement was set to a network size of 5 or participation in the prior month, some CDEs remained statistically significant and similar in magnitude to the TEs. Specifically, fixing “visiting friends/family,” “going out for enjoyment,” and “religious service attendance” to occurring in the prior month yields CDEs of −.405 (*p* < .001), −.404 (*p* < .001), and −.401 (*p* <.001), respectively. Notably, these CDEs are highly similar in magnitude to the corresponding TEs, indicating that educational differences in the risk of incident dementia changed little under conditions of prior month engagement in these social activities.

Three mediators showed weaker direct effects of education when social engagement is set to having occurred in the prior month. When volunteering is set to having occurred in the prior month, the CDE is considerably smaller in magnitude (CDE = −.144) and is statistically non-significant. Similarly, when organized activities are set to having occurred in the prior month, the CDE (−.221) is smaller than the TE and not statistically significant, as is the CDE for network size when set to 5 (CDE = −.312). Together, these results indicate that the protective influence of higher education on incident dementia generally persists when some forms of social engagement are reported as occurring in the prior month, but this direct effect is substantially attenuated and no longer statistically significant under conditions of volunteering and organized activities in the prior month, and with discussion networks of five members.

We next examined interaction and mediation. We did not find evidence of significant interactions between education and social engagement in their effects on incident dementia. However, visiting with friends/family, going out for enjoyment, volunteering, and organized group activity participation in the prior month each yielded statistically significant pure indirect effects, ranging from −.016 (*p* < .05) for visiting with friends/family to −.092 (*p* < .05) for organized group activities and −.093 (*p* < .01) for volunteering. Thus, even though the controlled direct effects of education were statistically significant even under conditions of visiting friends/family and going out for enjoyment in the prior month, these forms of social engagement are statistically significant pathways through which education shapes incident dementia.

**Figure 1:**
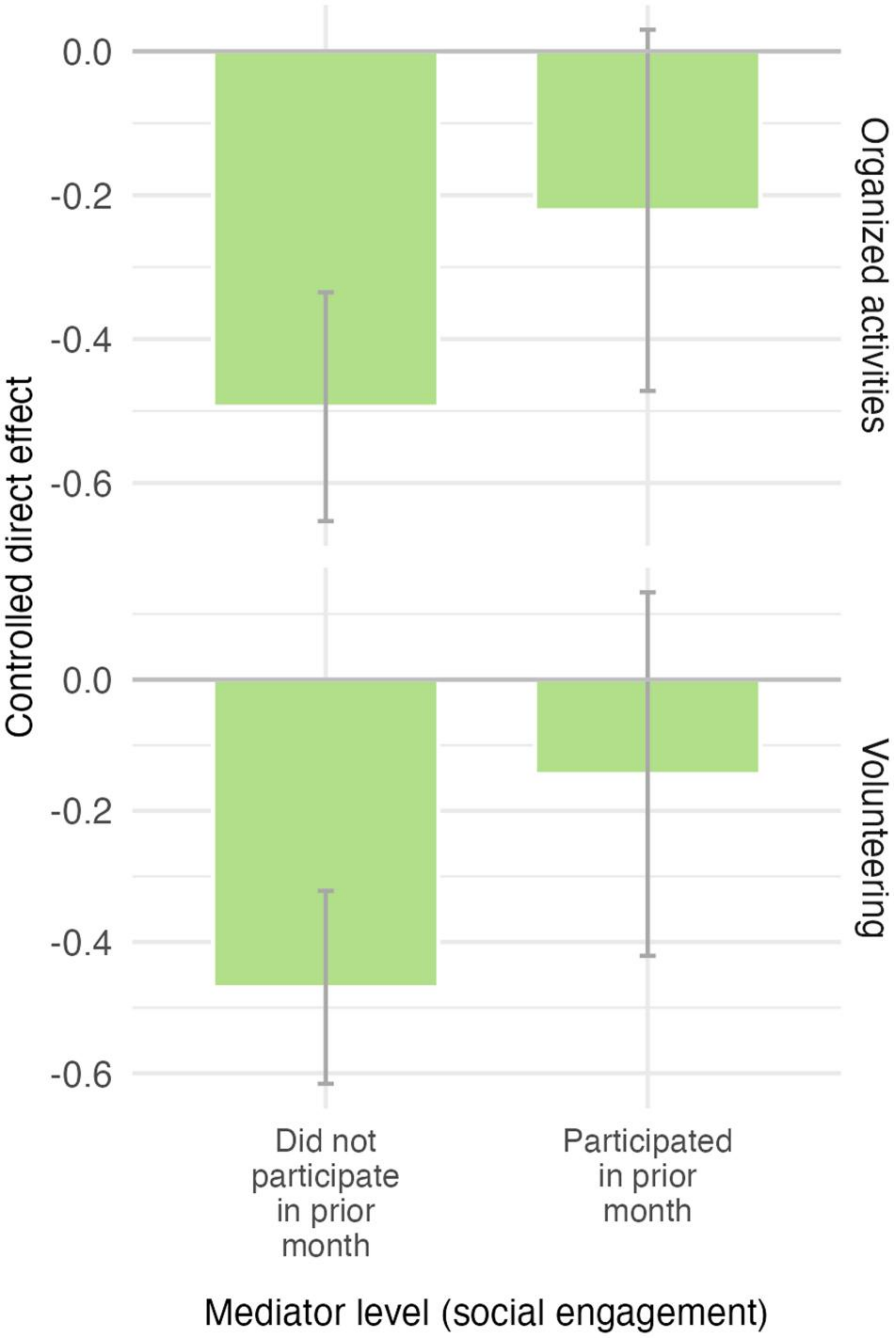
Controlled direct effects of education on incident dementia when social engagement levels (“Organized Activities” and “Volunteering”) are “Did not participate in the prior month” and “Participated in the prior month.”

In additional analyses, we compared the CDEs from models that estimated the effects of education on incident dementia in two hypothetical conditions. The first examined educational disparities under conditions in which respondents engaged in each social activity in the prior month (the same analyses as those presented in Table 4). The second examined educational disparities under conditions in which respondents did *not* engage in each social activity in the prior month. Figure 1 illustrates the CDEs from these decomposition analyses using organized group activities (top panel) and volunteering (bottom panel) as the mediators. The bars represent the excess relative risk of dementia that is associated with higher education (i.e., a Bachelor’s degree or higher) compared to having lower levels of education (i.e., a high school degree or less) when the social engagement mediator is fixed at *not* having participated in the prior month (left) and, separately, when it is fixed at having participated in the prior month (right).

The results illustrate that under conditions in which older adults across all levels of education did not engage in volunteering and organized group activity in the prior month, having a Bachelor’s degree was associated with significantly lower excess relative risks of incident dementia compared to those with a high school degree or less (CDE = −.469, *p* < .001 for volunteering; CDE = −.494, *p* < .001 for organized group activities). These estimated excess relative risks were smaller in magnitude and no longer statistically significant in models that set volunteering and organized group activity engagement to having occurred in the prior month, with CDEs of −.144 (*p* = .308) and −.221 (*p* = .085), respectively.

## Discussion

Research on dementia risk has long emphasized the protective role of education and, more recently, the importance of social engagement in later life (e.g., Livingston et al., 2024; Lövdén et al., 2020). Yet these literatures have largely developed in parallel, with limited integration of how early-life educational experiences and later-life social connections might jointly shape dementia risk. As a result, we know much about the independent associations of education and social engagement with cognitive aging, but far less about how these pathways intersect across the life course to influence inequalities in dementia. Without considering their intersection, however, we risk overlooking how enduring educational inequalities may shape the capacity of social ties to protect against dementia, and how late-life social engagement may either mitigate or exacerbate these disparities.

Our results documented educational gradients across forms of social engagement, with older adults with higher levels of education participating more recently (“in the prior month”) and having larger discussion networks. Using a counterfactual mediation approach with survival analyses, we found that educational disparities in incident dementia may be attenuated and no longer statistically significant under hypothetical scenarios where all respondents volunteered in the prior month, participated in organized group activities in the prior month, and had five discussion network members. The large and statistically significant associations between education and incident dementia remained in cases of visiting with friends and family, going out for enjoyment, and attending religious services in the prior month. Visiting with friends and family, going out for enjoyment, volunteering, and participation in organized group activities also emerged as significant indirect pathways through which education shapes incident dementia over 8 years of older adulthood. It is worth noting that the majority of respondents reported visiting with friends and family (87%), going out for enjoyment (79%), and attending religious services (60%) in the prior month, suggesting caution around concluding that engagement in these social activities does not reduce educational differences in incident dementia. Rather, more specific measurements of these activities in future research (e.g., spending time with friends outside of the home, going out for enjoyment in a new social setting) would allow for a more refined examination of the circumstances under which these general categories of social activity could influence the education–dementia association.

Although additional research on underlying mechanisms is needed, patterns in our mediation findings may reflect that activities such as going out for enjoyment, volunteering, and organized group activities often require individuals to leave the home to participate, more so than more intimate personal connections involved in visiting with friends and family and discussion network members. Exposures encountered outside of the home during social activity participation, including the built and social environmental features of older adults’ neighborhoods, benefit cognitive function as sources of environmental enrichment and cognitive stimulation (e.g., Finlay et al. 2022). More so than engagement with closer social ties, volunteering, going out for enjoyment, and organized group activities may foster social interaction with less familiar and more diverse social ties, which can benefit cognitive function and memory through exposure to novel social and environmental stimuli and contribute to cognitive reserve (Perry et al., 2022). Additionally, activities like volunteering may protect against dementia by promoting older adults’ engagement in complex and novel tasks, which benefit cognitive functioning (Proulx et al., 2018). Volunteering and other group activities can also promote life satisfaction (Van Willigen, 2000) or a sense of meaning or purpose, which is strongly predictive of lower dementia risk (Sutin et al., 2023).

Mediating pathways also encompass associations between education and each of these forms of social activity. Indeed, involvement in volunteer work and organized clubs during college and post-graduate educational experiences, and the occupational contexts that follow, may foster life-long interests in these forms of social engagement (e.g., Oesterle et al., 2004). Higher education is associated with more time spent engaging in physical activity (Flood & Moen, 2015), higher engagement in personal leisure and civic/religious activities (Morrow-Howell et al., 2014), as well as greater arts and cultural engagement, which may in part reflect financial and transportation access (Fluharty et al., 2021). Less educated older adults may remain in the labor force for longer at older ages, and be engaged in caregiving and other familial obligations that preclude other forms of social engagement.

It is important to consider that the NHATS is generalizable to the U.S. Medicare population aged 65 and older, but is not generalizable to the community-based population. The NHATS continued to follow and interview respondents as they transitioned into different residential settings (i.e., residential care facilities, nursing homes) and used proxy reports for those unable to respond themselves. In this sense, our study is inclusive of diverse residential settings and health conditions, but may also include respondents who differ in their opportunities for social engagement as a result of their residential context, and who transition out of the community-dwelling context as a result of significant physical and cognitive impairments–transitions that may also intersect with socioeconomic factors (Chyr et al., 2020). Supplemental analyses revealed that respondents who had at least one proxy report during the observation period had significantly lower levels of all forms of social engagement and a higher likelihood of dementia. Decomposition results were consistent when excluding proxy reports, but result consistency may differ among a population with significant health impairments. Indeed, Supplementary Table 1 reveals that physical and mental health indicators are associated with significantly lower social engagement and significantly higher hazards for incident dementia. A key direction for future research will be to examine whether mediating effects of social engagement on the education-dementia relationship differ among community-based versus other residential contexts, and among older adults who experience different health trajectories. Indeed, residential care and nursing home facilities may vary in accessible opportunities for residents’ social engagement, just as neighborhood communities and social environments also vary, in ways that have implications for cognitive function and dementia (e.g., Goldman, Finlay, et al., 2025).

### Additional considerations and limitations

Social engagement measures in the NHATS are limited in their ability to allow for a more detailed examination of social participation frequency and form. The dichotomous ‘yes/no’ response format prevents further distinguishing, for example, daily or weekly participation from once monthly participation or among less frequent engagement (e.g., a few times a year versus never). Likewise, because the social participation questions refer to “in the prior month,” we are unable to ascertain whether responses reflect longer-term, regular participation trends or monthly fluctuations that could be influenced by weather, illness, or other temporary life circumstances. Categories of social activity, such as “organized group activities,” are also broad in scope and may be interpreted differently among respondents, thus introducing measurement heterogeneity. Other datasets that collect more nuanced social participation information and detailed activity descriptions may shed further light on how specific types and frequencies of social participation over longer periods mediate and interact with education to shape incident dementia. Further research is needed to design, test, and implement interventions that increase volunteering and organized group activity engagement. Public health initiatives promoting these activities are also likely to benefit other dimensions of well-being that reduce dementia risk, such as physical activity, stress reduction, and positive mental health. Our estimates may be conservative, as they do not account for other pathways through which changes in social activity may reduce other dementia risk factors patterned by education (Perry, McConnell, Coleman, et al., 2022).

Unmeasured confounders—including experiences and exposures between the completion of education and later life—are sources of bias and limit causal inference. Although we restricted our sample to respondents who were dementia-free at baseline, selection bias may persist if higher levels of social engagement earlier in life contributed to both survival to age 65 and older, and to cognitive function at baseline. The cognitive assessment in the NHATS screens for dementia but cannot infer a diagnosis or disease stage. Since dementia pathology often begins before clinical symptoms appear (Amieva et al., 2008), the sample likely includes cases of dementia that are not detected by survey-based screenings. Survey-based cognitive assessments may also be educationally biased (e.g., Borda et al. 2019).

This study brings two key social determinants of cognitive aging into conversation while adding important nuance to prior research that has examined general indices of social isolation as potentially mediating education-cognitive function associations (Goldman, Wroblewski, et al., 2025). Future work could explore whether educational disparities in certain dimensions of cognition (e.g., memory, attention, executive function) are more or less mediated by different forms of social engagement. Comparisons using different measures of educational attainment (e.g., years of education) should also be examined. Whereas our analyses focused on comparisons between “less than high school” and “Bachelor’s degree or higher,” supplemental decomposition analyses (Supplementary Table 2) revealed that the magnitude of total and controlled direct effects declined incrementally when comparing higher levels of education (“high school or equivalent” and “some college”) with “Bachelor’s degree or higher,” although controlled direct effects remained nonsignificant for volunteering and organized group activities.

As high school and college completion rates in the United States have increased since the 1960s, future research should examine potential cohort effects. The role of social engagement in mediating education–dementia associations may be changing amidst a changing educational landscape. Variations across age and racial/ethnic groups should also be explored, given increases in educational attainment among Black and Hispanic Americans and among later-born cohorts (Garcia et al., 2021; Hayward et al., 2021). Finally, additional studies should examine the time-varying nature of social engagement in the years prior to dementia. In robustness checks, we found similar results when using mean values of social engagement, modeling the mediating effects of consistent monthly social engagement across rounds of observation. However, given that declines in cognitive function may influence levels of social engagement (e.g., Casey et al., 2021), and that cognitive decline may occur over years prior to detection on a survey-based dementia screening, additional research is needed to better understand the bidirectional and longitudinal dynamics of social engagement and cognitive function in the context of educational differences.

Finally, our findings carry implications for place-based social initiatives. Accessible opportunities that encourage older adults to leave their homes and engage socially may be an especially important means of protecting against or delaying dementia onset. Special attention should be paid to making these social activities accessible to older adults with lower levels of education, who may reside in neighborhoods with fewer opportunities for engagement.

## Supplementary Material

igag051_Supplementary_Data

## Data Availability

Data is publicly available through nhats.org (NHATS data). This study was not preregistered.
