# Supplementary material for: Is social connection the key to prevention? Educational attainment, incident dementia, and the role of later-life social engagement
Source: Innov Aging. 2026 May 4;10(7):igag051. doi: 10.1093/geroni/igag051 (PMC13294448; doi:10.1093/geroni/igag051)
Supplement: igag051_Supplementary_Data [file igag051_supplementary_data.pdf]

Innovation in Aging **Supplementary Material: Goldman, Cha, & Tayari Ashtiani. Is social connection the key to prevention? Educational disparities in dementia risk and the role of later-life social engagement.**

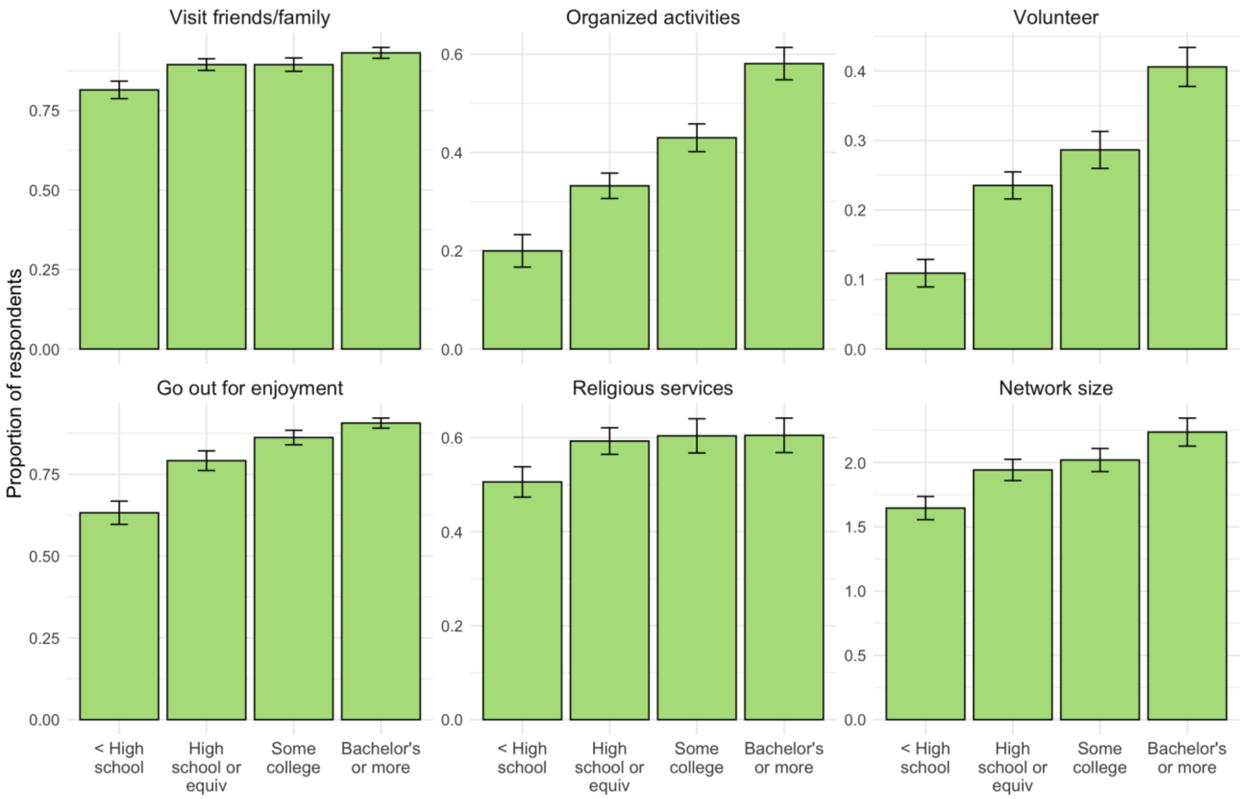

Supplementary Figure 1. Social Engagement by Educational Attainment in the NHATS.

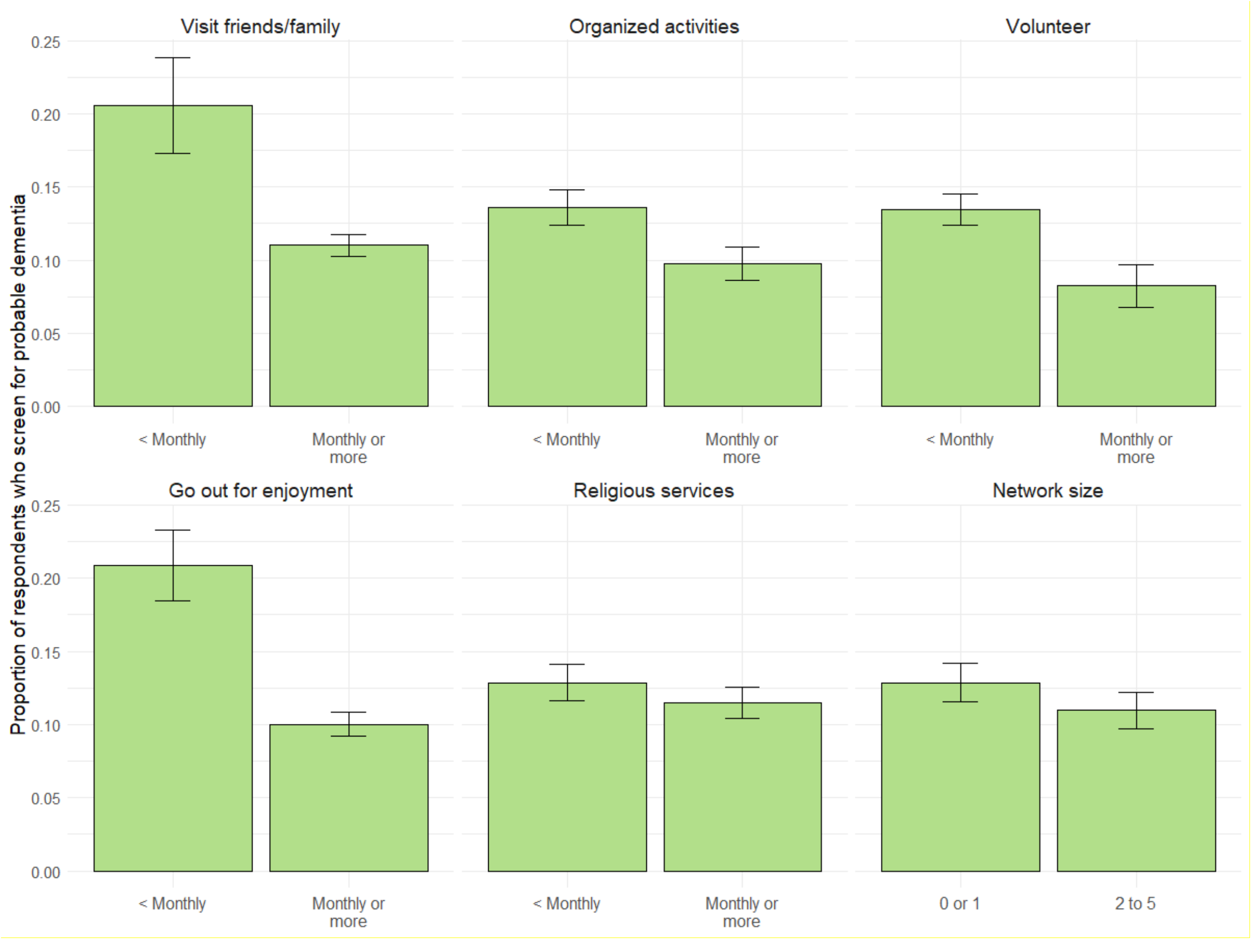

Supplementary Figure 2. Social Engagement by Dementia Status in the NHATS.

Supplementary Table 1. Coefficients from the multivariable regression analyses using the NHATS that provide the parameters for the four-way decomposition analysis, including (1) dementia as a function of education, the social engagement mediator, and covariates, and (2) social engagement as a function of education and covariates.

Variable	Social engagement mediator: Network size	Visit friends/ family	Go out for enjoyment	Volunteer	Organized activities	Religious services
**Model for the outcome (dementia-free survival)** [a]						
Education	.851*	.841*	.877	.823***	.812***	.824***
	(.055)	(.065)	(.059)	(.034)	(.036)	(.044)
Social mediator	.914	.698	.826	.542*	.659*	.855
	(.067)	(.136)	(.146)	(.139)	(.137)	(.143)
Education x	.995	.985	.941	1.17	1.100	1.006
social mediator	(.027)	(.083)	(.073)	(.101)	(.085)	(.068)
Age	1.096***	1.092***	1.092***	1.093***	1.094***	1.094***
	(.007)	(.006)	(.006)	(.006)	(.006)	(.006)
Female	.904	.853*	.850*	.841*	.848*	.852*
	(.076)	(.069)	(.068)	(.068)	(.068)	(.069)
Black	1.259**	1.246*	1.239*	1.270**	1.264**	1.310**
	(.111)	(.108)	(.108)	(.110)	(.109)	(.114)
Hispanic	1.625***	1.552**	1.547**	1.565**	1.577**	1.628***
	(.222)	(.210)	(.213)	(.213)	(.217)	(.223)
Married or living	.802**	.803**	.813*	.794**	.790**	.802**
with a partner	(.067)	(.065)	(.066)	(.065)	(.064)	(.065)
Worked for pay	.348***	.366***	.377***	.375***	.373***	.372***
recently	(.073)	(.073)	(.075)	(.075)	(.075)	(.074)
Self-rated	.837***	.846***	.849***	.849***	.842***	.839***
physical health	(.031)	(.031)	(.031)	(.031)	(.031)	(.031)
Depression	1.561***	1.508***	1.507***	1.522***	1.521***	1.539***
	(.154)	(.147)	(.146)	(.147)	(.147)	(.149)
Basic activities of	1.405***	1.390***	1.367***	1.385***	1.387***	1.386***
daily living	(.065)	(.063)	(.062)	(.062)	(.063)	(.063)
Currently smokes	1.085	1.059	1.066	1.059	1.061	1.053
	(.083)	(.080)	(.080)	(.080)	(.080)	(.080)
**Model predicting the mediator (social engagement)** [b]						
Education	.153***	.014***	.054***	.065***	.100***	.025***
	(.016)	(.004)	(.005)	(.005)	(.006)	.006
Age	-.005*	-.003***	-.003***	-.002*	.001	.002*
	(.002)	(.001)	(.001)	(.001)	(.001)	.001
Female	.559***	.054***	.044***	.024*	.093***	.134***
	(.035)	(.009)	(.011)	(.012)	(.013)	.013
Black	-.128**	-.048***	-.086***	-.005	-.027	.181***
	(.041)	(.011)	(.013)	(.014)	(.015)	.015
Hispanic	.149*	-.082***	-.120***	-.082**	-.044	.092**
	(.073)	(.019)	(.022)	(.024)	(.026)	.027
Married or living	.162***	.045***	.079***	.009	.018	.089***
with a partner	(.035)	(.009)	(.011)	(.012)	(.013)	.013
Worked for pay	.012	.009	.037*	.024	.060**	-.004
recently	(.050)	(.013)	(.015)	(.017)	(.018)	.019
Self-rated	.034*	.027***	.043***	.048***	.053***	.023***
physical health	(.016)	(.004)	(.005)	(.005)	(.006)	.006
Depression	-.048	-.044**	-.089***	-.053**	-.056**	-.029
	(.049)	(.013)	(.015)	(.016)	(.018)	.018

Variable	Social engagement mediator					
	**Network size**	**Visit friends/ family**	**Go out for enjoyment**	**Volunteer**	**Organized activities**	**Religious services**
Basic activities of daily living	.054	-.031***	-.073***	-.039***	-.043***	-.064***
	(.029)	(.007)	(.009)	(.009)	(.010)	.010
Currently smokes	-.077*	-.008	.000	-.020	-.023	-.083***
	(.033)	(.008)	(.010)	(.011)	(.012)	.012
N	6,339	6,459	6,458	6,461	6,461	6,461

Note. Robust standard errors appear in parentheses.

[a] Coefficients from Cox regression models are hazard ratios predicting incident dementia from 2012-2018.

[b] Coefficients are beta coefficients from linear regression models.

*** $p < .001$; ** $p < .01$; * $p < .05$ (Two-sided tests).

Supplementary Table 2. Total effects and controlled direct effects from four-way decompositions comparing "Bachelors' degree or more" with other levels of educational attainment.

	**Less than HS vs. BA or more (main results)**		**HS or equivalent vs. BA or more**		**Some college vs. BA or more**	
**Social engagement mediator**	Total effect (TE)	Controlled direct effects (CDE)	Total effect (TE)	Controlled direct effects (CDE)	Total effect (TE)	Controlled direct effects (CDE)
**Friends**	-.437*** (.063)	-.405*** (.069)	-.318*** (.051)	-.295*** (.053)	-.175*** (.031)	-.162*** (.031)
**Enjoy**	-.442*** (.062)	-.404*** (.074)	-.326*** (.050)	-.293*** (.055)	-.182*** (.031)	-.161*** (.032)
**Volunteer**	-.444*** (.062)	-.144 (.141)	-.312*** (.052)	-.114 (.106)	-.164*** (.033)	-.066 (.060)
**Organized activities**	-.440*** (.062)	-.221 (.128)	-.306*** (.054)	-.172 (.095)	-.158*** (.036)	-.098 (.053)
**Religious attendance**	-.439*** (.062)	-.401*** (.088)	-.320*** (.051)	-.293*** (.064)	-.175*** (.031)	-.161*** (.036)
**Network size**	-.428*** (.064)	-.312 (.160)	-.302*** (.052)	-.226* (.104)	-.171*** (.031)	-.123* (.052)

Note. Standard errors appear in parentheses. Estimates represent excess relative risks.
****p* < .001; ***p* < .01; **p* < .05 (Two-sided tests).
